# Species Richness and Composition of Forest Birds in Urban Parks and Reserves of Buenos Aires City, Argentina

**DOI:** 10.3390/ani14040602

**Published:** 2024-02-12

**Authors:** Ianina N. Godoy, Fabricio C. Gorleri, Maximiliano A. Cristaldi, Lucas M. Leveau

**Affiliations:** 1 Departamento de Ecología, Genética y Evolución, Facultad de Ciencias Exactas y Naturales, Universidad de Buenos Aires—Instituto de Ecología, Genética y Evolución de Buenos Aires, Ciudad Universitaria, Pab 2, Piso 4, Buenos Aires 1426, Argentina; ianinagodoy@hotmail.com (I.N.G.); maximilianocristaldi@yahoo.com.ar (M.A.C.); 2 Laboratorio de Ecología, Comportamiento y Sonidos Naturales, Instituto de Bio y Geociencias del Noroeste Argentino—Consejo Nacional de Investigaciones Científicas y Técnicas, Avenida 9 de Julio 14, Rosario de Lerma 4405, Argentina; fabriciogorleri@gmail.com

**Keywords:** area, citizen science, insectivorous birds, Latin America, native trees, urbanization

## Abstract

**Simple Summary:**

Urban green spaces provide multiple services and are important for biodiversity and human well-being. Nevertheless, studies analyzing the relationship between green spaces and forest birds in the Global South are still scarce. Using citizen science data, we evaluated the variation in the richness and specific composition of forest birds in two types of urban green spaces in the Metropolitan Area of Buenos Aires, Argentina: parks and reserves. Sampling effort was considered as the number of checklists for each site. Forest bird species richness was higher in reserves and was positively related to sampling effort. Forest bird species composition varied as a function of green space type and sampling effort. Moreover, the species present in sites with lower richness were a subset of the species present in richer sites. Reserves and sites with the highest sampling effort concentrated all species. The results obtained show the importance of urban reserves and citizen science platforms in the conservation of forest birds.

**Abstract:**

Urbanization is among the main factors of ecosystem transformation and threats to global biodiversity. Urban green spaces provide multiple services, being important for biodiversity and human well-being. However, the relationship between green spaces and forest birds has been scarcely studied in the Global South. In this work, we used citizen science data (eBird) to assess the variation in the species richness and composition of forest birds in two types of public urban green spaces characterized by different vegetation composition and management: parks and reserves. In general, reserves had more native and unmanaged vegetation than parks. We selected parks and reserves located in the coastal area of the Metropolitan Area of Buenos Aires, Argentina. Sampling effort was considered as the number of checklists for each site. The database allowed information to be extracted from 12 sites and 33 species. The most common species were the Green-barred Woodpecker (*Colaptes melanochloros*), the Narrow-billed Woodcreeper (*Lepidocolaptes angustirostris*), and the White-crested Tyrannulet (*Serpophaga subcristata*). Bird species richness was higher in reserves than in parks and was positively related to sampling effort. The forest bird species composition varied according to the type of green area and sampling effort. Species composition showed a significant nestedness, with the least rich sites being a subset of species from the richest sites. Reserves and sites with the highest sampling effort concentrated all species. The results obtained show the importance of urban reserves in the conservation of forest birds.

## 1. Introduction

Urban biodiversity has gained importance in recent years, not only because of the negative impact generated by the expansion and densification of cities [1,2,3], but also due to the growing recognition of the fundamental role of urban areas in the conservation of global biodiversity [4,5]. Therefore, cities can be planned and managed to favor and diversify native flora and fauna [6,7,8].

Urban green spaces (UGSs) such as parks, vacant lots, private or public gardens, river or stream banks, and protected areas provide multiple benefits for biodiversity [4] and human well-being [9,10]. Trees present in these spaces can provide shelter and food and facilitate the dispersal of forest bird species, mainly for habitat specialists [11,12,13]. Forest birds may be particularly affected by urbanization due the scarcity of their main food items, such as arthropods [14], and the replacement of native trees by exotic species [15]. In addition, the potential of UGSs to provide diversity of habitats and microhabitats for birds may be limited by management and maintenance regimes that usually simplify the vegetation cover, whether by grass mowing, excessive pruning, or the removal of vegetation, dead matter, shrubby and spontaneous vegetation, and leaf litter [7,16,17].

The relationships between forest birds and UGSs have been scarcely documented in the scientific literature and mainly concentrated in the northern hemisphere [11,18,19,20]. These studies have found that forest bird richness was negatively affected by the level of urbanization surrounding forest fragments and positively related to the size of fragments [11,20,21,22]. In addition, the presence of unmanaged vegetation and native trees may favor the diversity and abundance of forest birds in UGSs [12,16,19,21]. The composition of forest birds has been associated with the surrounding urban cover, UGS size, and vegetation cover [11,18,20]. Moreover, forest bird assembly has shown a nested pattern [23], with smaller and more human-disturbed UGSs containing a subset of species of larger and less disturbed UGSs [11]. However, the relationships between UGSs and forest birds in Latin America have not been evaluated yet.

Due to the availability of bird data from large areas, citizen science data have been increasingly used to explore bird–habitat relationships in urban environments [24,25,26,27]. We used citizen science data to evaluate how the richness and composition of forest birds varies in UGSs with different vegetation management and size in the Metropolitan Area of Buenos Aires, Argentina. We expected that bird richness would be higher in larger UGSs and with the presence of unmanaged and native vegetation. In addition, we expected that bird composition would differ depending on the type of vegetation management and the size of the UGS. A better understanding of bird–habitat relationships would allow us to propose suitable management and urban planning measures in one of the most urbanized and, at the same time, one of the most biodiverse areas in the region [28].

## 2. Methods

### 2.1. Study Area

**Figure 1:**
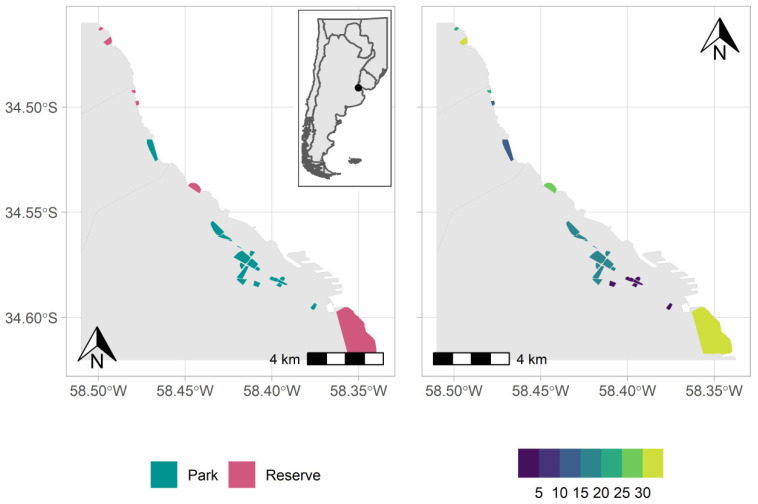
Location of the study area in Argentina (insert), parks and reserves in the Metropolitan Area of Buenos Aires (**left**), and forest bird species richness in the urban green spaces (**right**).

This study was carried out along a green corridor located in the districts of San Isidro, Vicente López, and Buenos Aires City, all of them belonging to the Metropolitan Area of Buenos Aires (MABA). The green corridor extends along the riverbank of the La Plata River [29] (Figure 1). The MABA is the most populated region in Argentina, with more than 15 million inhabitants [30]. It has a temperate climate, with cold and dry winters (July: 11 °C, 60.6 mm) and hot and rainy summers (January: 24.9 °C, 138.8 mm) (https://www.smn.gob.ar/estadisticas, accessed on 10 August 2022). It is located in an ecotone comprising the Rolling Pampa, belonging to the Pampean Phytogeographic Province, and the forests and deltas of the Paraná River, belonging to the Paranaenese Phytogeographic Province [31]. Due to this confluence of different environmental units, the native forest formations of the region are characterized by their structural diversity, from sparse woodlands to complex riparian forests, and by the formation of “neocommunities” dominated by invasive exotic trees, a product of urbanization [29].

### 2.2. Forest Birds

We considered forest birds to be those species whose diet consists of 50% or more invertebrates and that use more than 50% of the understory, middle strata, and canopy in forests to feed [32]. Although Wilman et al. [32] indicated that the diet of the Streaked Flycatcher (*Myiodynastes maculatus*) is composed of 40% invertebrates, other studies have recorded the species as predominantly insectivorous in urban environments [33,34]. Therefore, the species listed in Table 1 were considered for analyses. Species associated with environments close to water bodies were discarded.

### 2.3. eBird Data

Records of forest birds were obtained through the citizen science platform eBird Argentina. eBird was created in 2002 and contains bird observation data in checklist format, where detected species are seen or heard, by one or more volunteer observers, during a sampling event (https://ebird.org/about, accessed on 20 June 2022) [35]. It has a robust review process that allows for the correct identification of species prior to their incorporation into the database [36]. We collected information on the presence of forest birds in all uploaded checklists for Buenos Aires City, Vicente López, and San Isidro until April 2022, including all sampling protocols and all levels of sampling effort. The observations with displacement whose distance traveled was greater than the sampled perimeter as well as those that did not report all the species observed were excluded.

We identified the urban green spaces located in the coastal green corridor using Google Earth Pro and a vector layer of public green spaces downloaded from the public green spaces database of the Sub-Management of Spatial Information, Secretariat of Innovation and Digital Transformation (https://data.buenosaires.gob.ar/dataset/, accessed on 20 June 2022 ) (Figure 1). The data were created in May 2021 and are updated quarterly. The polygons of the sites located in the districts of Vicente López and San Isidro were delimited using Google Earth software version 7.3.

To obtain the presence records of the species at each site, we crossed the data downloaded from eBird with the polygons of the selected green spaces (see La Sorte et al. [25]). For the final selection of the sites, we prioritized those sites that had the greatest number and also selected an equal number of parks and reserves, resulting in a total of 8459 checklists and 12 sites. Because the number of checklists per site ranged from 16 to 5915, we included sampling effort as an additional covariate in the models.

### 2.4. Green Space Types

The selected urban green spaces were divided into parks and reserves according to vegetation management. Parks had a predominance of open, landscaped areas, with designed and highly managed vegetation that included irrigation, removal of dead matter, and frequent grass cutting, mostly of exotic origin [37,38]. The size of parks ranged between 6.39 ha and 91.22 ha (mean = 30.02 ha, *n* = 6). Due to the proximity of some selected parks, those located less than 200 m away from each other were analyzed as a single sample (Figure 1). On the other hand, reserves had protected native flora and fauna of the region, dominated by spontaneous tree, shrub, and herbaceous vegetation [39,40]. In general, vegetation management is limited to the control of exotic species [40,41,42,43]. Ecological reserves, municipal reserves, natural parks, and protected landscapes were included in this category. The size of reserves ranged between 2.87 ha and 317 ha (mean = 59.64 ha, *n* = 6).

### 2.5. Statistical Analysis

We defined species richness as the total number of forest bird species that were recorded and uploaded to the eBird platform for each site. We fitted generalized linear models (GLMs) to evaluate the relationships between the estimated species richness and green space type (park or reserve), area (ha), and sampling effort (measured in the number of checklists per site), using the glm function of the R Project program [44]. UGS size and sampling effort were logarithmically transformed prior to analysis. We assumed a Poisson distribution for species richness, with a logarithmic link function, and analyzed the over- and under-dispersion of the data. To remove the multicollinearity of predictor variables, we estimated the Pearson correlation coefficient and calculated generalized variance inflation factors using the gvif function of the glmtoolbox package in R Project [45]. Due to green space size and sampling effort being highly correlated (r = 0.75), we only included sampling effort in further analyses since this variable was the most correlated with species richness. The significance of the models was tested by comparing them with null models, using likelihood ratio tests (LRTs) with the anova function (*p* < 0.05).

We compared taxonomic composition between green space types and sampling effort using nonmetric multidimensional scaling (NMDS). NMDS is an ordination method that allows for visualizing relationship patterns between study units in a low-dimensional space, using dissimilarity data [46]. In this analysis, we use Jaccard’s coefficient as a measure of distance [47]. To ordinate the species and sites, the metaMDS function of the vegan package in R Project was used [48]. The variable types of green space and sampling effort were superimposed on the NMDS graph without altering the configuration of the original ordinations through the envfit function of the vegan package. As with the GLMs, we excluded the area of the sites due to its high correlation with sampling effort. Furthermore, we estimated the degree of nestedness through the NODF index, using presence–absence matrices. We tested the statistical significance of the index using the online software Nestedness for Dummies (NeD; https://ecosoft.alwaysdata.net/, accessed on 24 June 2023), by comparing the observed NODF with a null model [49,50] Strona et al., 2014) with 999 random matrices and 95% confidence intervals. The null model assigns to each matrix cell a probability to be occupied proportionally to the corresponding row and column totals [50]. Finally, we evaluated the roles of habitat type, sampling effort, and site area size on increasing nesting order through a nonparametric comparison of Mann–Whitney and Spearman correlations, using the wilcox.test and cor.text functions [44], respectively.

## 3. Results

The eBird database was composed of a total of 37 species, ranging between three and 18 for parks, and between 12 and 35 for reserves (Figure 1, Table 1). The most common species were the Green-barred Woodpecker (*Colaptes melanochloros*), the Narrow-billed Woodcreeper (*Lepidocolaptes angustirostris*), and the White-crested Tyrannulet (*Serpophaga subcristata*) (Table 1).

**Figure 2:**
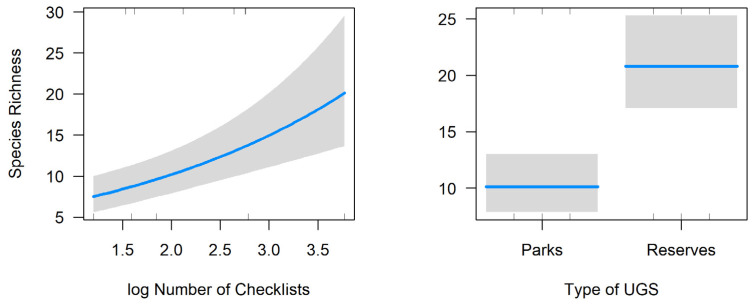
Relationships between species richness and the number of eBird checklists (**left**) and type of urban green space (UGS). The blue line represents the fitted line (**left**) or the mean values (**right**) and the gray areas, 95% confidence intervals.

Species richness was significantly associated with the type of green space and the amount of sampling effort (LRT = 60.25, df = 2, *p* < 0.001; Table 2). Species richness was higher in reserves and in sites with the highest sampling effort (Figure 2).

**Figure 3:**
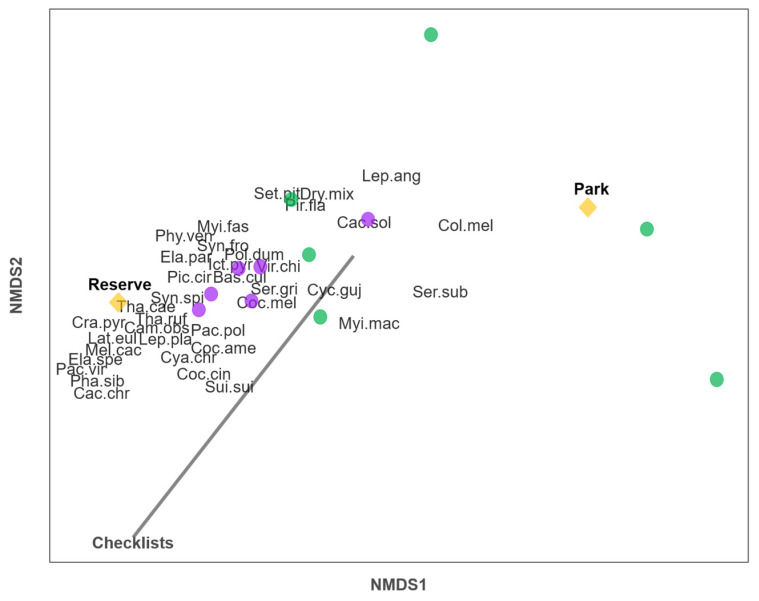
Nonmetric multidimensional scaling showing the relationships (*p* < 0.05) between forest bird species, green space types, and the number of eBird checklists. Violet circles represent reserves and green circles represent parks. The gray vector shows the direction of linear correlation between the number of eBird checklists (sampling effort) and ordination scores. Yellow diamonds indicate the centroids of parks and reserves. See species codes in Table 2.

The NMDS (stress = 0.054) showed that the Green-barred Woodpecker, the Narrow-billed Woodcreeper, and the White-crested Tyrannulet characterized the park communities, whereas the White-barred Piculet (*Picoides cirratus*), Euler’s Flycatcher (*Lathrotriccus euleri*), and the Rufous-capped Antshrike (*Thamnophilus ruficapillus*) characterized the reserve communities and the sites with a greater sampling effort (Figure 3).

**Figure 4:**
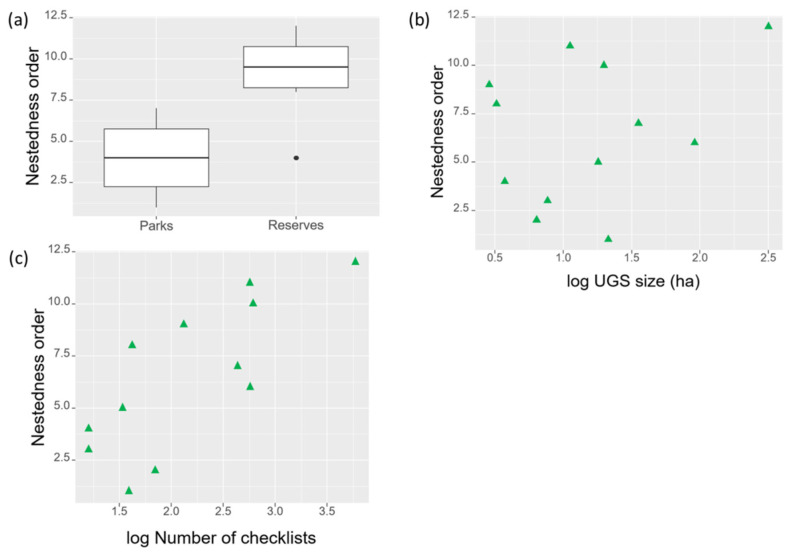
Relationships between the nestedness rank order of sites and (**a**) green space type, (**b**) green space size, and (**c**) the number of checklists. Higher rank indicates more species in sites. In (**a**), the central lines in the box plots represent the median, the hinges are the first and third quartiles (the 25th and 75th percentiles), and the whiskers are the largest and smallest values within 1.5 times the interquartile ranges below the 25% and above 75% percentiles.

Forest bird species composition was significantly nested (NODF = 89.81, *p* < 0.001), indicating that the species composition of poorer sites was a subset of the richest sites. Nestedness was related to the green space type, with species in parks being a subset of species in reserves (U = 3, *p* = 0.015, Figure 4a, Table 1). We did not find significant correlations between the nested rank of sites and green space size (r = 0.16, *p* = 0.62, Figure 4b), but a significant positive correlation with sampling effort (r = 0.75, *p* = 0.005, Figure 4c).

## 4. Discussion

Our results show that the urban reserves of the coastal green corridor maintained the highest richness of forest species and differed in composition from the parks located in the same corridor. Bird species recorded at the sites with the lowest species richness were subsets of those recorded at sites with the highest species richness.

The increase in bird species richness was related to habitat type, being higher in urban reserves. Reserves may offer more vegetation diversity, providing a greater variety of habitats and availability of resources for birds [19,51]. Although we did not quantify habitat diversity, several authors have described the presence of scrub and understory substrates in the reserves [39,40]. A greater vegetation diversity is related to greater species richness in urban environments [52,53,54,55,56,57,58] and, in particular, forest birds [11,19,54,59,60,61]. Some authors have found that the diversity of vegetation strata and invertebrate biomass are factors that are closely related with and jointly influence the richness of forest birds [59].

Higher species richness in reserves could be linked to the predominance of native vegetation in this type of urban green space. Several studies have shown the importance of native vegetation for the diversity of urban birds [7,57,62,63], particularly for riparian species [64], forest birds [51,61], and insectivorous birds [12,65,66]. On the other hand, the availability of dead matter, which is usually removed from parks for aesthetic and safety reasons, is key for some forest birds that nest in holes, such as woodpeckers [4,67]. Finally, leaf litter is another factor that could increase the availability of resources for forest birds [68].

Green space size was also positively associated with forest bird species richness. Species–area relationships have been widely addressed in studies of urban environments [25,60,69,70,71], mostly indicating that patch size is one of the main factors influencing variation in species richness. Larger areas are expected to have greater environmental heterogeneity and resource availability, thus supporting more species than smaller green spaces [58,70,71,72]. On the other hand, larger areas can support larger bird populations, thus decreasing local extinction rates [70,73,74].

Forest bird assemblages varied in composition depending on habitat type and sampling effort. Understory vegetation, which is almost removed in most urban parks [4], is an important source of resources for both forest birds [13] and insectivores [12]. For example, species associated with reserves such as the Bran-colored Flycatcher (*Myiophobus fasciatus*), Euler’s Flycatcher, the Sooty-fronted Spinetail (*Synallaxis frontalis*), and the Spix’s Spinetail (*Synallaxis spixi*) (Figure 3) largely depend on shrub vegetation for feeding and nesting [75,76,77]. On the other hand, more sampling effort may allow the detection of more species.

Spatial bias towards protected areas is a common occurrence when using citizen science data. Many volunteer observers prefer to view these types of areas or those with high species diversity for their records [36]. This disparity in the data can limit the construction of complex statistical models or analysis of other measures of diversity when comparing bird communities from different UGSs. However, our data show that, regardless of the habitat type, larger areas had more species lists. The increased sampling effort in larger areas is expected in studies analyzing species–area relationships [78]. On the other hand, citizen science data are heterogeneous regarding the identification skills of participants [79]. Therefore, our results should be taken with caution, especially for those similar species such as *Elaenia* sp. and *Serpophaga* sp.

Moreover, the species assemblages presented a nested pattern that was related to the type of green space, with the least rich sites (parks) being subsets of the richest sites (reserves). In addition, the rank of sites was positively related to sampling effort. On the one hand, our results agree with those obtained by Wang et al. [80] because habitat type probably determined the nested pattern of species. Urban reserves could have certain habitat features, such as shrubs, a dense understory, and native tree species that allow rare and specialist bird species to thrive. On the other hand, passive sampling could be another factor determining the nested pattern of species [80], because more sampling may allow for recording rare species in comparison to less surveyed sites where only common species were detected. Other factors such as patch isolation and human disturbance were not measured in our study and could play a role in determining species nestedness in parks and reserves [80,81].

## 5. Conclusions

Our results highlight the importance of reserves for urban biodiversity regardless of their size in the coastal green corridor of the Metropolitan Area of Buenos Aires. Future studies should include environmental variables not analyzed in this study—such as vegetation composition and structure and human disturbance—that can improve our understanding of the effects of habitat type on forest bird communities. However, we consider that our results provide valuable information for public space managers and support the importance of the usage of citizen science platforms in ecological studies conducted in urban environments.

## Figures and Tables

**Table 1:** List of forest bird species recorded in urban reserves and parks of the Metropolitan Area of Buenos Aires, Argentina. The last two columns on the right indicate the number of sites in which species were recorded in reserves (*n* = 6) and parks (*n* = 6).

English Name	Scientific Name	Code	Reserves	Parks
Ash-colored Cuckoo	*Coccycua cinerea*	Coc.cin	3	1
Dark-billed Cuckoo	*Coccyzus melacoryphus*	Coc.mel	4	2
Yellow-billed Cuckoo	*Coccyzus americanus*	Coc.ame	1	0
White-barred Piculet	*Picumnus cirratus*	Pic.cir	4	1
White-fronted Woodpecker	*Melanerpes cactorum*	Mel.cac	1	0
Checkered Woodpecker	*Dryobates mixtus*	Dry.mix	6	4
Green-barred Woodpecker	*Colaptes melanochloros*	Col.mel	6	6
Rufous-capped Antshrike	*Thamnophilus ruficapillus*	Tha.ruf	3	0
Variable Antshrike	*Thamnophilus caerulescens*	Tha.cae	4	0
Narrow-billed Woodcreeper	*Lepidocolaptes angustirostris*	Lep.ang	6	5
Tufted Tit-Spinetail	*Leptasthenura platensis*	Lep.pla	3	0
Freckle-breasted Thornbird	*Phacellodomus sibilatrix*	Pha.sib	1	0
Stripe-crowned Spinetail	*Cranioleuca pyrrhophia*	Cra.pyr	3	0
Spix’s Spinetail	*Synallaxis spixi*	Syn.spi	5	0
Sooty-fronted Spinetail	*Synallaxis frontalis*	Syn.fro	6	1
Green-backed Becard	*Pachyramphus viridis*	Pac.vir	1	0
White-winged Becard	*Pachyramphus polychopterus*	Pac.pol	5	1
Mottle-cheeked Tyrannulet	*Phylloscartes ventralis*	Phy.ven	5	2
Large Elaenia	*Elaenia spectabilis*	Ela.spe	2	0
Small-billed Elaenia	*Elaenia parvirostris*	Ela.par	4	1
Suiriri Flycatcher	*Suiriri suiriri*	Sui.sui	2	1
Southern Beardless-Tyrannulet	*Camptostoma obsoletum*	Cam.obs	4	0
White-crested Tyrannulet	*Serpophaga subcristata*	Ser.sub	6	5
Straneck’s Tyrannulet	*Serpophaga griseicapilla*	Ser.gri	6	2
Streaked Flycatcher	*Myiodynastes maculatus*	Myi.mac	5	3
Bran-colored Flycatcher	*Myiophobus fasciatus*	Myi.fas	6	1
Euler’s Flycatcher	*Lathrotriccus euleri*	Lat.eul	3	0
Rufous-browed Peppershrike	*Cyclarhis gujanensis*	Cyc.guj	5	2
Chivi Vireo	*Vireo chivi*	Vir.chi	6	3
Plush-crested Jay	*Cyanocorax chrysops*	Cya.chr	2	1
Masked Gnatcatcher	*Polioptila dumicola*	Pol.dum	6	3
Solitary Black Cacique	*Cacicus solitarius*	Cac.sol	6	2
Golden-winged Cacique	*Cacicus chrysopterus*	Cac.chr	2	0
Variable Oriole	*Icterus pyrrhopterus*	Ict.pyr	5	3
Tropical Parula	*Setophaga pitiayumi*	Set.pit	5	4
Golden-crowned Warbler	*Basileuterus culicivorus*	Bas.cul	5	3
Hepatic Tanager	*Piranga flava*	Pir.fla	6	4

**Table 2:** Results of the generalized linear model for the forest bird richness as a function of habitat type (urban parks (intercept) and reserves) and sampling effort (log of eBird number of checklists).

Variable	Estimate	Std. Error	z	*p*
Intercept	1.555	0.221	7.050	<0.001
Reserve	0.720	0.160	4.497	<0.001
Number of Checklists (log)	0.383	0.087	4.396	<0.001

## Data Availability

The datasets generated and/or analyzed during the current study are available upon request to the corresponding author.
